# Thalamo-Nucleus Accumbens Projections in Motivated Behaviors and Addiction

**DOI:** 10.3389/fnsys.2021.711350

**Published:** 2021-07-15

**Authors:** Aurélie De Groote, Alban de Kerchove d’Exaerde

**Affiliations:** Laboratory of Neurophysiology, ULB Neuroscience Institute, Université Libre de Bruxelles (ULB), Brussels, Belgium

**Keywords:** thalamus, nucleus accumbens, goal-directed, reward, addiction

## Abstract

The ventral striatum, also called nucleus accumbens (NAc), has long been known to integrate information from cortical, thalamic, midbrain and limbic nuclei to mediate goal-directed behaviors. Until recently thalamic afferents have been overlooked when studying the functions and connectivity of the NAc. However, findings from recent studies have shed light on the importance and roles of precise Thalamus to NAc connections in motivated behaviors and in addiction. In this review, we summarize studies using techniques such as chemo- and optogenetics, electrophysiology and *in vivo* calcium imaging to elucidate the complex functioning of the thalamo-NAc afferents, with a particular highlight on the projections from the Paraventricular Thalamus (PVT) to the NAc. We will focus on the recent advances in the understanding of the roles of these neuronal connections in motivated behaviors, with a special emphasis on their implications in addiction, from cue-reward association to the mechanisms driving relapse.

## Introduction

The nucleus accumbens (NAc), a key node of the reward brain circuitry, is known to be involved in several motivated behaviors and is notably responsible for translating motivation into action in goal-directed behaviors (Mogenson et al., 1980; Ikemoto and Panksepp, 1999; Carelli, 2002; Klawonn and Malenka, 2018; Yang et al., 2018). The NAc is also part of the circuits that support drug addiction (Belin and Everitt, 2008; Yager et al., 2015; Everitt and Robbins, 2016; Scofield et al., 2016), a chronic and relapsing disorder characterized by the continuation of drug use despite harmful consequences (Leshner, 1997; Goodman, 2008). Drugs of abuse hijack normal adaptive changes in the brain that occur in a non-pathological context to drive reward-related learning and memory, thereby involving the NAc (Nestler, 2013).

The NAc receives glutamatergic projections from cortical and thalamic areas in similar abundance (Phillipson and Griffiths, 1985; Doig et al., 2010). These projections innervate the main neuronal populations of the striatum, the D1- and D2-Medium Spiny Neurons (MSNs) as well as interneurons (Smith et al., 2004; Doig et al., 2010; Wall et al., 2013; Klug et al., 2018; Johansson and Silberberg, 2020). Although thalamic afferents of the NAc have long been overlooked, recent studies have untangled their functional importance.

The thalamus is composed of the principal (or “relay”) sensorimotor nuclei, the “association” nuclei, and the midline and intralaminar thalamic nuclei (Groenewegen and Witter, 2004; Vertes et al., 2015). Thalamic projections to the NAc are mainly concentrated in this last group, and more precisely in the paraventricular thalamic nucleus (PVT) and in the central medial nucleus (Su and Bentivoglio, 1990; Vertes and Hoover, 2008; Vertes et al., 2015). Given the growing evidences highlighting its key role in various motivated behaviors, the PVT is the main focus of this mini-review. Importantly, the PVT is a critical hub for homeostatic and internal state information, and is likely to convey this information to the NAc to influence goal-directed responses to homeostatic challenges (Penzo and Gao, 2021).

The PVT is subdivided into two parts which differ in terms of inputs (Li and Kirouac, 2012), outputs, and cellular composition (Gao et al., 2020). The anterior PVT (aPVT) innervates preferentially the dorsomedial NAc shell, while the posterior PVT (pPVT) sends projections mainly to the ventromedial NAc shell (Dong et al., 2017). PVT neurons projecting to the NAc core are less numerous than to the NAc shell, but can be found throughout the anterior-posterior extent of the PVT (Dong et al., 2017). Importantly, PVT connections are highly conserved across species, even though the strength of connections may differ in specific structures (Hsu et al., 2014). However, the distinction between the aPVT and pPVT in human is difficult to identify (Hsu et al., 2014).

Notably, the PVT also stimulates the release of dopamine in the NAc by making close contact with dopamine terminals (Pinto et al., 2003; Parsons et al., 2007; Kirouac, 2015; Perez and Lodge, 2018). Other mechanisms of thalamic-dependent dopamine release cannot be excluded, since the thalamus is able to activate striatal cholinergic interneurons to drive local dopamine release (Threlfell et al., 2012; Kosillo et al., 2016; Johnson et al., 2017; Cover et al., 2019).

## Thalamo-Nucleus Accumbens Projections: Rewarding or Aversive?

Given the important role of the NAc in motivated behaviors and the relevance of the PVT in negative emotional behaviors (Hsu et al., 2014; Kirouac, 2021), several groups have tried to assess the motivational valence of the PVT-NAc pathway (PVT = > NAc) (Table 1). In a real-time place preference assay (RTPP), mice seemed to reduce the time spent in the chamber associated with the optogenetic stimulation of PVT = > NAc shell (Zhu et al., 2016; Do-Monte et al., 2017). This aversion seemed to be dependent on glutamatergic transmission in the NAc, since the avoidance of the optogenetic stimulation was abolished by AMPA receptor (AMPAR) antagonist in the NAc, but not by D1 or D2 antagonists (Zhu et al., 2016). Another paper, however, showed no preference or avoidance of the optogenetic stimulation chamber, highlighting instead the high variability of behaviors during this RTPP (Lafferty et al., 2020). Indeed, some mice avoided the stimulation zone, others increased the time spent in that zone, and a third group had no preference or aversion but a high transition rate between both zones. Interestingly, in an operant chamber, acute PVT = > NAc stimulation contingent to lever press induced self-stimulation, suggesting that brief synchronous activation of these projections is reinforcing rather than aversive (Lafferty et al., 2020). Finally, a recent study showed that the precise stimulation of aPVT neurons expressing corticotrophin-releasing factor (aPVT^CRF^) and projecting to the NAc shell induced behavioral aversion in the RTPP (Engelke et al., 2021).

**TABLE 1:** Summary of the principal studies directly investigating thalamo = > accumbens projections in various motivated behaviors.

	References	Projections	Behaviors	Methods	Results
Homeostatic and reward-seeking behaviors	Labouèbe et al., 2016	PVT (*Slc2a2*) = > NAc	Sucrose-seeking	Optogenetic stimulation (terminals)	↑ Motivated sucrose seeking
	Do-Monte et al., 2017	aPVT = > NAc (mainly shell)	Sucrose-seeking	Optogenetic inhibition (terminals)	↑ Sucrose-seeking during reward omission
				Optogenetic stimulation (terminals)	↓ Sucrose-seeking
			RTPP	Optogenetic stimulation (terminals)	Behavioral aversion
	Cheng et al., 2018	aPVT = > NAc	Novelty-suppressed feeding task	Optogenetic stimulation (terminals)	↑ Feeding
	Ren et al., 2018	PVT = > NAc	Sleep	Optogenetic stimulation (terminals)	↑ Transitions from sleep to wakefulness
				Chemogenetic inhibition (cell bodies)	↓ Wakefulness
	Meffre et al., 2019	pPVT = > NAc core	Sucrose-seeking	Optogenetic stimulation (terminals)	↑ NAc core neuronal responses to reward-predictive cues in sated animals
	Otis et al., 2019	PVT = > NAc	Pavlovian conditioning	*In vivo* two-photon calcium imaging of PVT neurons projecting to the NAc	Inhibitory responses to reward-predictive cues.
	Lafferty et al., 2020	PVT = > NAc shell	Operant task with cued periods of reward unavailability	Optogenetic inhibition (cell bodies or terminals)	↑ Unproductive reward seeking
			Operant task without periods of reward unavailability	Chemogenetic activation (cell bodies)	↓ Reward-seeking
			RTPP	Optogenetic stimulation (terminals)	High variability
			Self-stimulation	Optogenetic stimulation (terminals)	Reinforcing
	Christoffel et al., 2021	aPVT = > NAc	Limited-access high fat exposure	Optogenetic stimulation (terminals)	↑ High fat intake during acquisition period
				Optogenetic inhibition (terminals)	↓ High fat intake during acquisition and expression periods
				Chemogenetic inhibition (cell bodies)	↓ High fat intake during expression period
				Optogenetic stimulation and brain slice electrophysiology	↑ AMPAR/NMDAR ratio at aPVT = > D1R-MSNs synapses
				Optical LTD induction protocol *in vivo*	↓ High fat intake after optical LTD
			High fat CPP	Optogenetic inhibition (terminals)	↓ High fat paired chamber preference
			Operant task: progressive ratio (high fat pellets)	Chemogenetic inhibition (cell bodies)	↓ Breakpoint
	Engelke et al., 2021	aPVT = > NAc	Conflict test (food and predator odor)	Chemogenetic inhibition (cell bodies)	↓ Defensive responses ↑ Food-seeking behavior
		aPVT^CRF^ = > NAc shell	Conflict test (food and predator odor)	Optogenetic stimulation (terminals)	↓ Food-seeking
			RTPP	Optogenetic stimulation (terminals)	Behavioral aversion
Drug experience and addiction	Joffe and Grueter., 2016	Midline thalamic nuclei = > NAc core	Cocaine exposure followed by 2 weeks of abstinence	Optogenetic stimulation and brain slice electrophysiology	↑ AMPAR and NMDAR function at D1R-MSNs ↑ Silent synapses at D2R-MSNs
	Neumann et al., 2016	PVT = > NAc shell	Cocaine self-administration	Disruption of synaptic transmission (tetanus toxin)	↓ Acquisition of cocaine self-administration
			1–2 days of withdrawal after cocaine self-admin.	Optogenetic stimulation and brain slice electrophysiology	↑ Silent synapses ↑ Presynaptic release probability
			45 days of withdrawal after cocaine self-admin.		↑ Presynaptic release probability
	Zhu et al., 2016	PVT = > NAc medial shell	RTPP	Optogenetic stimulation (terminals)	Behavioral aversion
			Naloxone-precipitated opiate withdrawal	Optogenetic inhibition (terminals) or optogenetic long term depression protocol (terminals)	↓ Somatic signs of opiate dependence ↓ Place-aversion
			CPA induced by spontaneous opiate withdrawal/mild footshock/LiCl intraperitoneal injection	Chemogenetic inhibition (terminals) before CPA conditioning	↓ Place-aversion
		PVT = > NAc	Chronic morphine exposure	Optogenetic stimulation and brain slice electrophysiology	↑ AMPAR/NMDAR ratio in D2R-MSNs
	Wunsch et al., 2017	Anterior midline and intralaminar thalamic nuclei = > NAc	Reinstatement of cocaine self-administration after extinction	Chemogenetic inhibition (cell bodies)	↑ Cue-induced reinstatement ↓ Drug-primed reinstatement
	Keyes et al., 2020	PVT = > NAc	Morphine CPP	Chemogenetic inhibition (terminals) or optogenetic inhibition (terminals) during CPP expression	↓ Retrieval of opiate associated memories (persistently) ↓ Morphine-primed relapse
	Chisholm et al., 2021	PVT = > NAc shell	Relapse after heroin self-administration followed by withdrawal and food restriction	Chemogenetic activation (terminals)	↓ Heroin seeking in food-restricted rats

*All methods presented here directly targeted the thalamic neurons projecting to the NAc, either by retrograde transport of viral tools from the NAc to the thalamus (cell bodies), or by local manipulation of the thalamic projections in the NAc (terminals). CPA, conditioned place aversion; CPP, conditioned place preference; CRF, corticotrophin-releasing factor; LTD, long-term depression; NAc, nucleus accumbens; PVT, paraventricular thalamus; aPVT, anterior paraventricular thalamus; pPVT, posterior paraventricular thalamus; RTPP, real-time place preference. Arrow up means increased. Arrow down means decreased.*

Overall, the neuronal activity pattern in the PVT seems critical in determining the rewarding or aversive properties of this pathway: brief optogenetic stimulations being possibly reinforcing and prolonged stimulations aversive. Moreover, even though all the studies presented here focused on the PVT = > NAc shell, the precise shell subregions targeted could explain the discrepancies in the results. Indeed, NAc subregions differentially modulate motivational valence, highlighting distinct functions of the circuits along the medio-lateral axis of the shell (Yang et al., 2018), its dorso-ventral axis (De Jong et al., 2019; Yuan et al., 2019) as well as its rostro-caudal axis (Reynolds and Berridge, 2002).

Finally, if the mixed effects of PVT = > NAc stimulation on motivational valence contrast with the rewarding effects of other glutamatergic inputs to the NAc (Stuber et al., 2011; Britt et al., 2012), they however seem to share interesting commonalities with glutamatergic inputs stimulation from the ventral tegmental area (VTA) (Qi et al., 2016; Yoo et al., 2016; Zell et al., 2020). Indeed, brief stimulation of VTA glutamatergic neurons induces positive reinforcement, in opposition to a more sustained stimulation which elicits apparent behavioral avoidance (Yoo et al., 2016).

## Reward-Seeking Behaviors

In addition to being an essential relay in wakefulness through a lateral hypothalamus = > PVT = > NAc circuit, (Ren et al., 2018), the PVT also integrates other homeostatic information to influence reward seeking behaviors, including reward consumption and feeding behaviors. A population of PVT glutamatergic neurons expressing the glucose transporter Glut2 (*Slc2a2*) and projecting to the NAc has been identified. These neurons, controlled by glucose metabolism, are activated by hypoglycemia conditions and mediate sucrose-seeking (Labouèbe et al., 2016). A recent study also revealed the role of aPVT = > NAc in promoting high fat intake, and shed light on the potentiation of aPVT synapses with NAc D1-MSNs after repeated exposure to the high fat reward (Christoffel et al., 2021). Interestingly, aPVT = > NAc seemed to regulate the incentive motivational value of the diet rather than hunger itself, since inhibition of this circuit decreased the preference for a high fat paired chamber, as well as the breakpoint in a progressive ratio task (Christoffel et al., 2021).

Several lines of evidence also suggest a key role of PVT circuitry in adaptive behavior, by influencing the decision to seek or avoid a reward (McGinty and Otis, 2020). A study showed that during behavioral competition between reward and danger, PVT inhibition induced a bias in the behavior, depending on the experimental conditions, toward either reward or defense (Choi and McNally, 2017; Choi et al., 2019), confirming the dual role of PVT = > NAc in aversion and reward. Another study showed that the activation of aPVT = > NAc neurons increased feeding behavior in a novel environment, without altering anxiety behaviors (Cheng et al., 2018). Recently, aPVT^CRF^ = > NAc has also been shown to control the conflict between approach-food and avoid-predator threat by suppressing food-seeking in the presence of a predator odor (Engelke et al., 2021). It is likely that the PVT = > NAc modulation during conflict also strongly depends on the internal state of the animal as well as on the precise experimental conditions, possibly explaining (in addition to the precise cellular identity of the neurons targeted in the aPVT) the discrepancies between these last two studies.

All these studies seem to indicate a crucial role of PVT = > NAc in coordinating relevant homeostatic inputs, including arousal and hunger information, to drive context-dependent reward-seeking behaviors (Table 1). Notably, the PVT is extensively innervated by hypothalamic peptides, such as orexin (Kelley et al., 2005; Kirouac et al., 2005), the neuropeptide Y and cocaine- and amphetamine-regulated transcript, that are likely to contribute to the integration of arousal, feeding, and reward seeking behaviors (Adamantidis and de Lecea, 2009; Choi et al., 2012; Mahler et al., 2014; Sakurai, 2014; Baimel et al., 2015; Lee et al., 2015). Finally, direct connections of hypothalamic orexin neurons with the VTA and the NAc may also play a role in these behaviors, and have been reviewed elsewhere (Haghparast et al., 2017).

Cue-reward association and salience processing are important components of reward seeking behaviors. PVT neurons are activated in response to cues associated with food reward (Choi et al., 2010; Igelstrom et al., 2010), and influence the attribution of incentive values to reward cues (Haight et al., 2015, 2017, 2020; Campus et al., 2019). Moreover, both the midline thalamic nuclei (MTN) and the NAc receive prefrontal cortex (PFC) inputs that are able to modulate conditioned reward seeking through divergent cue encoding in PFC neurons (Otis et al., 2017). Fiber photometry experiments were performed to characterize associative learning in the PVT, and showed that PVT neurons are activated by reinforcing stimuli and predictive cues (Zhu et al., 2018). The PVT responded to cues and stimuli regardless of palatable or aversive outcome, but the intensity of their response reflected the magnitude of the reward or punishment. In addition, PVT responses were modulated by behavioral homeostatic states and were associated with the salience of sensory stimuli in a context-dependent manner. Optogenetic inhibition also demonstrated that PVT responses during the predictive cue and/or the reinforcing stimulus were necessary for reward seeking, aversive learning and extinction (Zhu et al., 2018). Another study also confirmed the involvement of PVT neurons in different aspects of cue-induced motivated behavior (Munkhzaya et al., 2020). Finally, PVT neurons also encode reward omission (Do-Monte et al., 2017; Zhu et al., 2018). All these studies highlight the key roles played by thalamic neurons in salience processing (Zhou et al., 2021).

Several groups studied specifically the involvement of PVT = > NAc in cue-reward association and reward-seeking (Table 1). In a cue-induced sucrose-seeking task, omission of the reward during cue-on periods usually leads to an increased reward-seeking response. Photoinhibition of aPVT = > NAc resulted in an even further increase in the reward-seeking during the periods of reward omission, while optogenetic activation of aPVT = > NAc decreased the cue-induced pressing for sucrose (Do-Monte et al., 2017). In a similar study, photoinhibition of PVT = > NAc increased unproductive reward seeking during reward unavailability periods, while pharmacological activation of PVT = > NAc decreased operant responding during a task in which reward was always available (Lafferty et al., 2020). Both studies highlighted the implications of PVT = > NAc in adapting the reward seeking behavior to the context and reward availability.

To better understand how reward-seeking relevant inputs are integrated by PVT = > NAc neurons, mice were trained to associate a conditioned stimulus with a sucrose reward (Otis et al., 2019). Calcium imaging revealed that more than half of tracked PVT = > NAc neurons developed an inhibitory response to the conditioned stimulus across learning (Otis et al., 2019). However, electrophysiological recordings demonstrated that both NAc core and pPVT neurons were excited by reward-predictive cues (Meffre et al., 2019). This discrepancy could result from the different population of PVT neurons, or from the precise NAc regions targeted, the NAc shell and core being especially implicated in feeding behavior and incentive-cue responding, respectively (Stratford and Kelley, 1999; Ambroggi et al., 2011). Interestingly, the hunger state of the animal modulated these neuronal responses in the NAc core and pPVT, and orexin transmission in the pPVT increased sucrose-seeking and NAc core neuronal responses to the cues (Meffre et al., 2019). This suggests the existence of a orexin neurons = > pPVT = > NAc core circuit that conveys energy-balance information to drive cue responses in this sucrose-seeking task (Meffre et al., 2019), highlighting again the involvement of these projections in processing homeostatic inputs.

Despite some contradictions, probably due to the precise neuronal targeting, all these papers demonstrate the crucial role of PVT = > NAc in integrating and processing reward-relevant inputs to drive appropriate goal-directed behaviors. Importantly, some discrepancies across laboratories in the directionality of the effect of PVT = > NAc stimulation or inhibition may also be explained by the internal states (such as arousal and metabolic state) or past experience information integrated by the PVT.

## Drug Experience and Addiction

Interestingly, these reward processing mechanisms can also be hijacked by drugs of abuse. The PVT has been known to be involved in several aspects of drug-induced behaviors, including the locomotor response to psychostimulants (Young and Deutch, 1998; Clark et al., 2017), cue-reward association (Brown et al., 1992), expression of drug-induced place preference (Browning et al., 2014), withdrawal (Smith et al., 2020), and reinstatement of drug-seeking (James et al., 2010). The roles of thalamus in addiction have been reviewed elsewhere (Huang et al., 2018).

Several studies shed light on changes occurring specifically at thalamo = > NAc synapses after drug of abuse experience (Table 1). Cocaine experience was shown to enhance AMPAR and NMDA receptor (NMDAR) functions at the synapses between the MTN and D1-MSNs in the NAc core. The altered NMDAR properties most likely resulted from the incorporation of GluN2C/D-containing NMDARs. The cocaine exposure also generated silent synapses between the MTN and D2-MSNs (Joffe and Grueter, 2016). Similarly, another study showed that one day of cocaine self-administration increased the level of AMPAR-silent PVT = > NAc shell synapses, likely by the insertion of GluN2B-containing NMDARs. In addition, the presynaptic release probability was increased. After withdrawal, silent synapses returned to basal levels, probably through the insertion of non-calcium-permeable AMPARs. In contrast, the basal presynaptic release probability of these synapses between the PVT and NAc shell was persistently increased after cocaine exposure (Neumann et al., 2016). Lastly, a third study showed that escalating morphine regimen increased AMPAR/NMDAR ratio at PVT = > NAc synapses in D2- but not D1-MSNs, probably by the insertion of calcium-permeable AMPARs (Zhu et al., 2016). Different drugs of abuse thus alter these thalamo = > NAc synapses at short and long term, through both pre- and post-synaptic mechanisms.

Several groups have also begun to decipher the functional and behavioral importance of PVT = > NAc in various aspects of addiction (Table 1). Disruption of synaptic transmission between the PVT and the NAc demonstrated that PVT = > NAc was necessary for cocaine self-administration (Neumann et al., 2016). In addition, inhibition of PVT = > NAc disrupted the expression of a morphine conditioned place preference (CPP) (during and one day after the inhibition), and prevented morphine-primed relapse (Keyes et al., 2020). Moreover, inhibition of the PVT caused a disinhibition of NAc = > lateral hypothalamus and the blockade of the CPP retrieval, highlighting a PVT = > NAc = > lateral hypothalamus pathway important for the retrieval and maintenance of opiate-associated memories (Keyes et al., 2020).

PVT = > NAc also mediates the aversive symptoms of opiate withdrawal, as photoinhibition of PVT = > NAc medial shell reduced the aversive effects of both naloxone-induced and spontaneous opiate withdrawal (Zhu et al., 2016). This effect was not specific to opiate withdrawal since these projections were also necessary for mild footshock and injections of LiCl to evoke behavioral aversion (Zhu et al., 2016). With regards to relapse, inhibition of the MTN (including the PVT) and intralaminar thalamic nuclei during the reinstatement of cocaine seeking reduced both cue-induced and drug-primed reinstatement, but did not affect the reinstatement of sucrose-seeking (Wunsch et al., 2017). Interestingly, specifically decreasing the activity of the anterior MTN = > NAc neurons increased cue-induced but reduced drug-primed reinstatement of cocaine seeking, suggesting different mechanisms by which MTN = > NAc neurons modulate these two types of relapse (Wunsch et al., 2017).

Finally, two studies also linked drug-seeking behavior to the hunger state of the animal, by showing that chronic food-restriction increased heroin-seeking behavior after withdrawal from self-administration. This phenomenon was modulated by PVT = > NAc shell neurons, as their enhanced activity attenuated this food restriction-induced heroin seeking (Chisholm et al., 2020, 2021), demonstrating an overlap between PVT = > NAc circuits driving food- and drug-seeking behaviors.

All these studies highlight a key role of PVT = > NAc in various aspects of drug-induced behaviors, from self-administration and conditioned drug seeking to the negative states induced by withdrawal, and relapse.

## Discussion and Conclusion

In this mini-review, we have highlighted the latest research advancements toward understanding the roles of thalamo = > NAc projections (mainly PVT = > NAc) in various aspects of motivated behaviors, including reinforcement, reward-seeking and addiction. These PVT = > NAc projections, depending on their activity pattern, seem to be able to drive both aversive and reinforcing behaviors. A growing amount of evidence also showed a specific role of these afferents in various homeostatic behaviors, including arousal and feeding, as well as in cue-reward association. Moreover, drug exposure has been shown to induce both short- and long-term modifications at thalamo = > NAc synapses, and these projections have been demonstrated to play key roles in drug-seeking, withdrawal and relapse. Despite the increasing quantity of papers investigating the functions of these thalamo = > NAc projections, more studies will be necessary to fully understand their precise roles in motivated behaviors and addiction. Moreover, the cellular diversity of thalamo = > NAc neurons, the inputs influencing their activity and their precise encoding of reward-relevant information still remain to be fully deciphered. Finally, future studies with good spatial resolution and precise distinctions between the aPVT and pPVT as well as between the different regions of the NAc are likely to contribute to reconciling some contradictory findings of the recent literature.

## Author Contributions

AD and AK wrote the manuscript and edited and contributed to the final version of the manuscript. AK supervised all aspects of the work. Both authors contributed to the article and approved the submitted version.

## Conflict of Interest

The authors declare that the research was conducted in the absence of any commercial or financial relationships that could be construed as a potential conflict of interest.

## Abbreviations

AMPA: α-amino -3-hydroxy-5-methyl-4-isoxazolepropionic acid
AMPAR: AMPA receptor
aPVT: anterior paraventricular thalamus
CPA: conditioned place aversion
CPP: conditioned place preference
CRF: corticotrophin-releasing factor
D1: dopamine receptor D_1_
D2: dopamine receptor D_2_
GABA: γ-aminobutyric acid
MSN: medium spiny neuron
MTN: midline thalamic nuclei
NAc: nucleus accumbens
NMDA: *N*-methyl -D-aspartate
NMDAR: NMDA receptor
PFC: prefrontal cortex
pPVT: posterior paraventricular thalamus
PVT: paraventricular thalamus
PVT = > NAc: paraventricular thalamus neurons projecting to nucleus accumbens
RTPP: real-time place preference
SPN: striatal projection neuron
VTA: ventral tegmental area.
